# Assessment of Salivary Malondialdehyde and Superoxide Dismutase Levels in Completely Edentulous Patients: An In Vivo Study

**DOI:** 10.7759/cureus.27949

**Published:** 2022-08-12

**Authors:** SRN Venkata Harish V, Sriharsha Pudi, Rajasekhar Reddy Gade, Srinivas Vudi, Venkata Kanaka Dinesh Kumar BN, Sai Seshadri Bharath Thota

**Affiliations:** 1 Department of Prosthodontics and Crown & Bridge, Ganni Subba Lakshmi (GSL) Dental College & Hospital, Rajahmundry, IND; 2 Department of Prosthodontics, Crown & Bridge, MNR Dental College and Hospital, Hyderabad, IND; 3 Department of Prosthodontics and Crown & Bridge, St. Joseph Dental College and Hospital, Eluru, IND; 4 Department of Prosthodontics and Crown & Bridge, Anil Neerukonda Institute of Dental Sciences, Visakhapatnam, IND; 5 Department of Prosthodontics and Crown & Bridge, Drs. Sudha & Nageswara Rao Siddhartha Institute of Dental Sciences, Vijayawada, IND; 6 Department of Orthodontics, Narayana Dental College and Hospital, Nellore, IND

**Keywords:** malondialdehyde (mda), complete dentures, superoxide dismutase (sod), saliva, residual ridge resorption

## Abstract

Introduction

Residual ridge resorption (RRR) is a chronic inflammatory process; the free radicals formed may lead to tissue damage in the form of bone resorption mediating through many pathways and processes. We aimed to study the correlation between levels of malondialdehyde (MDA) and superoxide dismutase (SOD) enzyme and residual ridge resorptive status levels in completely edentulous patients.

Methods

This study included 45 patients aged 40-65 yrs who were completely edentulous. The mean levels of MDA and SOD were evaluated and correlated with different classes of bone resorption, period of edentulism, the effect of denture wearing, age and gender.

Results

The mean value of MDA levels in completely edentulous patients was 2.6 ± 1.23 and that for SOD was 1.8 ± 0.47. There was no statistically significant difference in MDA levels whereas SOD levels showed statistical significance between all four classes (p<0.05). MDA levels showed statistically significantly higher levels in patients with an edentulism period of one year or less and non-denture wearers. There was no statistically significant difference in MDA and SOD levels in relation to age and gender.

Conclusion

Within the limitations of the study, there may be higher antioxidant activity in patients with more resorption. The present study correlates with other studies on RRR done using radiographs and other clinical parameters implicating that MDA and SOD may be used as biomarkers of RRR.

## Introduction

Residual ridge resorption (RRR) is a term used to describe changes that affect the alveolar ridge following tooth extraction and continue even after healing of the extraction socket. RRR is a complex process that occurs irrespective of whether the patient wears a denture or not. A study by Bergman and Carlsson showed that resorption varies among individuals and within the same individual at different time periods. Individuals with higher rates of bone resorption initially tend to show more resorption in future [1]. According to Lammie, if the patient does not wear a denture, the resorption is attributed to disuse atrophy, an atrophying mucosa seeking a reduced area, thereby causing pressure resorption of the ridge [2]. The resorption of the denture wearer can be due to forces from the denture to ridge causing resorption.

Free radicals (FR) are defined as “any species capable of independent existence that contains one or more unpaired electrons”. Free radicals cause tissue damage by many mechanisms like DNA damage, lipid peroxidation, protein damage, oxidation of important enzymes and stimulation of pro-inflammatory cytokines. As RRR is a chronic inflammatory process, the free radicals formed may lead to tissue damaging in the form of bone resorption mediating through many pathways and processes and one of the processes is lipid peroxidation. The defence system of the body acts on the free radicals either to eliminate, scavenge or remove them. The imbalance between reactive oxygen species (ROS) or FR formed and the antioxidant defence system of the body creates oxidative stress [3]. Biomarkers of the antioxidant defence system include enzymatic antioxidants like superoxide dismutase (SOD), glutathione peroxidase (GPx) and catalase, and non-enzymatic antioxidants like uric acid, vitamin C and vitamin E. Biomarkers of free radicals depend on the type of mechanism that causes the tissue damage. These biomarkers help us to evaluate the extent and type of injury that occurred following the inflammatory phenomenon; hence, they give an indication of the extent and progress of the disease process [4,5].

Superoxide dismutase is an antioxidant enzyme that is present within the cell and protects it. Three isoenzymes of SOD are seen in humans: Cu/Zn SOD, present in cytoplasm and nucleus; Mn-SOD, present in mitochondria and EC-SOD, present extracellularly. Its main function is to remove damaging ROS from the cellular environment by catalysing the dismutation of O2- to H2O2. As lipid peroxidation is an outcome of oxidative stress, tissue damage is caused in the form of bone resorption. Malondialdehyde (MDA) is the main end product of the lipid peroxidation process [6]. The present study evaluates the levels of MDA and SOD in completely edentulous patients and correlates their levels with different classes of bone resorption, period of edentulism, the effect of denture wearing, age and gender.

## Materials and methods

Patient selection and sample size calculation

This cross-sectional study was conducted at the Department of Prosthodontics, Saveetha Dental College, Chennai. The sample size was calculated using 80% power and 95% confidence interval utilizing the nMaster sample size calculation software (Christian Medical College, Vellore, India). The sample size was found to be 42 and to overcome the errors during evaluation, it was rounded off to 45. Patients aged 40-65 yrs who were completely edentulous for 0-10 yrs were included in the study. Patients with systemic diseases, those with conditions like gout and arthritis, with smoking habit and alcoholics were excluded. This study was approved by the Scientific Ethical Review Board, Saveetha University, Chennai. All patients were informed about the study and informed consent was obtained.

**Figure 1:**
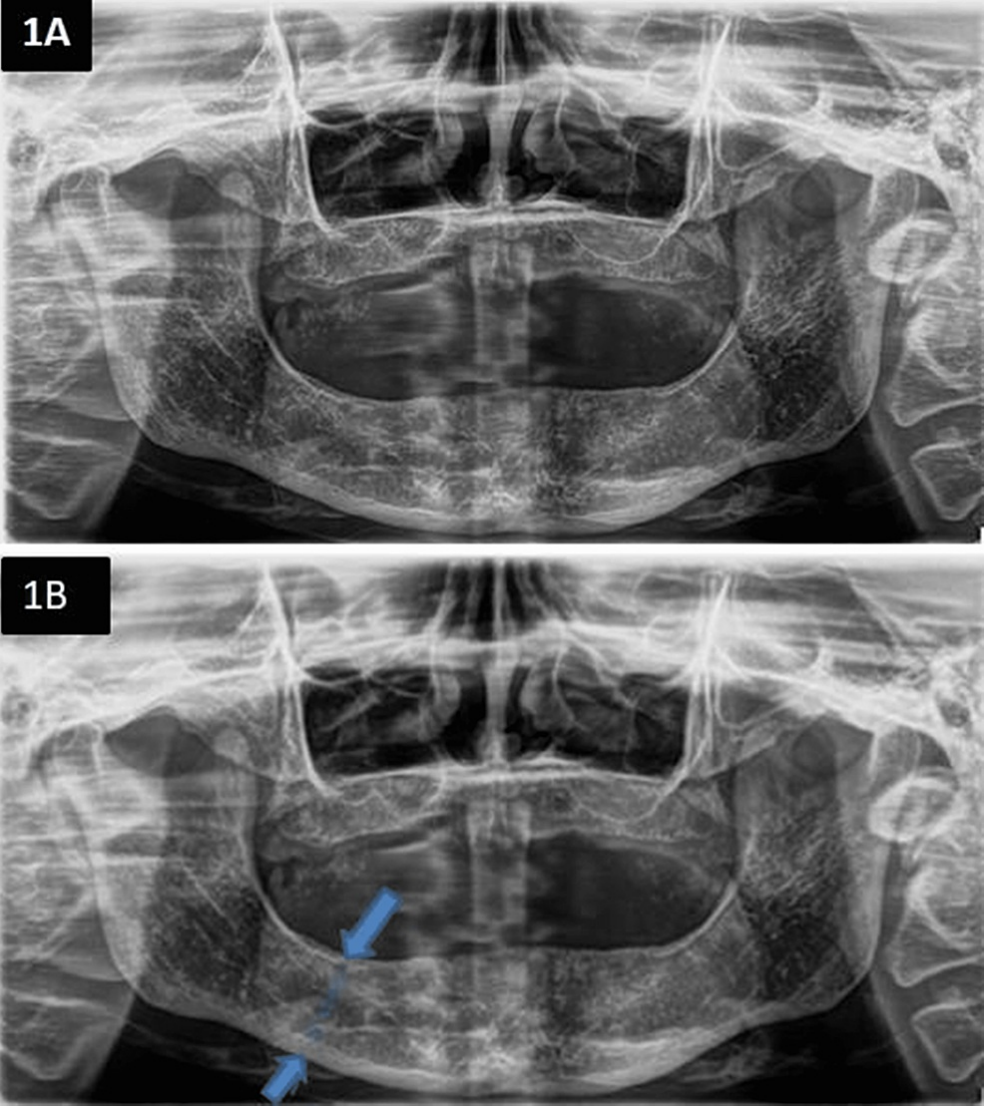
(A-B) Pre-operative orthopantomogram of the patient, with the bone height measured (arrows) using CorelDRAW Graphics Suite X6

A proforma for each patient was filled and an orthopantomogram (OPG) was taken for each (Figure 1A). The proforma includes mandibular bone height, maxillary residual ridge morphology, mandibular muscle attachments, maxilla mandibular relationship, conditions requiring pre-prosthetic surgery, interarch space and tongue anatomy, each criterion having its requirements to place under a class. Mandibular bone height was measured using CorelDRAW Graphics Suite X6 (Corel Corp., Ottawa) (Figure 1B). Based on the American Dental Association 1999 classification of edentulism, patients were divided into four classes: class I, ideal/minimally compromised; class II, moderately compromised; class III, substantially compromised; class IV, severely compromised [7].

Saliva collection

**Figure 2:**
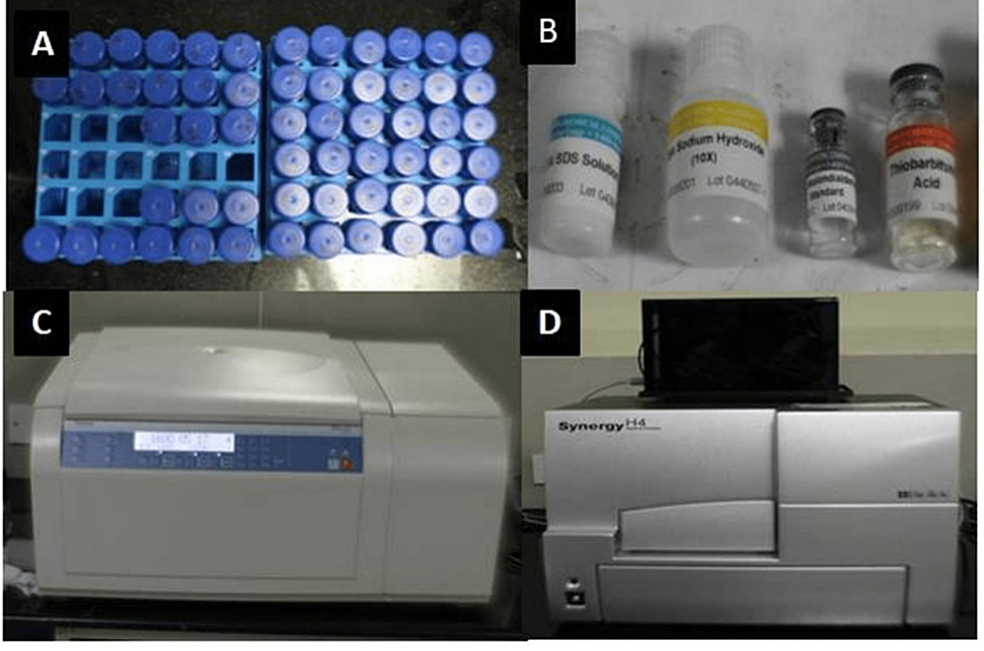
(A) Labeling of samples after collection, (B) TBARS assay kit components, (C) cooling centrifuge, (D) ELISA reader TBARS, thiobarbituric acid reactive substance; ELISA, enzyme-linked immunosorbent assay

The patients were asked not to have food for two hours before saliva collection. They were asked to rinse their mouths with distilled water. When they were in a relaxed state, 10 ml of unstimulated saliva was collected in sterile disposable test tubes by expectorating into the tubes for about five minutes (Figure 2A). Test tubes were then tightened with a screw cap, and centrifuged immediately at 2000 x g for 10 min at 4°C to remove cell debris. The supernatant was removed and stored at a low temperature (-8°C) until the assay was performed.

Determination of salivary MDA levels

MDA levels were determined using a thiobarbituric acid reactive substance (TBARS) assay kit (Cayman Chemical, Ann Arbor, MI) (Figure 2B). A total of 100 µl of sample was taken in a test tube using an Eppendorf pipette (Eppendorf SE, Hamburg) and 100 µl of the sodium dodecyl sulfate (SDS) solution and 4 ml of the colour reagent were added, following which the vials were capped. Then the vials were placed in a holder and kept in boiling water for one hour. After one hour, vials were removed and placed in an ice bath to stop the reaction and incubated on ice for 10 min. After that, the vials were centrifuged for 10 min at 1600 x g at 4°C (Figure 2C). Vials were kept stable at room temperature for 30 min. Then, 150 µl from each vial was loaded to the plate and the absorbance was read at 530-540 nm (Figure 2D).

Determination of salivary SOD levels

SOD levels were determined using the Misra and Fridovich method. It measures the enzymatic activity of SOD by auto-oxidation of epinephrine. This method uses SOD ability to inhibit autoxidation of epinephrine at alkaline pH. Since the oxidation of epinephrine leads to the production of pink adrenochrome, the rate of increase of the absorbance at 480 nm represents the rate of autoxidation of epinephrine (Figure 2D). It was observed that SOD inhibits this process of radical mediation. Then 0.5 ml of the saliva sample was treated with 0.5 ml of 1.8 mM epinephrine, 0.5 ml of 0.6 mM ethylenediaminetetraacetic acid (EDTA), and 0.50 ml of 0.3 M sodium carbonate (pH 10.2). The reaction was initiated by the addition of epinephrine, and the enzymatic activity was noted at an absorbance of 480 nm. One unit of SOD activity was defined as the concentration of the enzyme (mg protein/ml) in the plasma that caused a 50% reduction in the autoxidation of epinephrine.

## Results

The SD values of MDA and SOD levels in completely edentulous patients of different age groups are shown in Table 1. The Mann-Whitney test showed no statistically significant difference in MDA and SOD levels between each group (p<0.05) (Table 1).

**Table 1:** Mann-Whitney test with Bonferroni correction for pairwise comparison in relation to age MDA, malondialdehyde; SOD, superoxide dismutase p-value was measured by the Mann-Whitney test.

Variables	Age	N	SD	p-value
MDA levels	40-50 yrs vs. 51-60 yrs	11	1.26	0.350
	40-50 yrs vs. >60 yrs	18	1.22	0.438
	51-60 yrs vs. >60 yrs	16	1.42	0.916
	Total	45	1.30	
SOD levels	40-50 yrs vs. 51-60 yrs	11	0.47	0.672
	40-50 yrs vs. >60 yrs	18	0.54	0.642
	51-60 yrs vs. >60 yrs	16	0.38	0.999
	Total	45	0.46	

The mean value of MDA levels in completely edentulous patients with a period of edentulism ≤1 yr was 2.9 ± 1.14, >1 to <5 yrs was 2.5 ± 1.65 and >5 to 10 yrs was 1.7 ± 0.9. The mean value of SOD levels in completely edentulous patients with a period of edentulism ≤1 yr was 1.8 ± 0.48, >1 to <5 yrs was 1.8 ± 0.43 and >5 to 10 yrs was 1.9 ± 0.38. The Mann-Whitney test showed no statistical significant difference in MDA levels between the class with ≤1 yr vs. 1-5 yrs and class with 1-5 yrs vs. >5 yrs of edentulism period, but there was a statistical significant difference in the class with ≤1 yr vs. >5 yrs (p<0.05) of edentulism period. There was no statistical significant difference in SOD levels between class ≤1 yr vs. 1-5 yrs, class 1-5 yrs vs. >5 yrs and class ≤1 yr vs. >5 yrs (p<0.05) (Table 2).

**Table 2:** Mann-Whitney test with Bonferroni correction for pairwise comparison in relation to the period of edentulism MDA, malondialdehyde; SOD, superoxide dismutase

Variables	Period of edentulism	p-value
MDA levels	≤1 yr vs. 1-5 yrs	0.438
	1-5 yrs vs. >5 yrs	0.017
	≤1 yr vs. >5 yrs	0.269
SOD levels	≤1 yr vs. 1-5 yrs	0.749
	1-5 yrs vs. >5 yrs	0.856
	≤1 yr vs. >5 yrs	0.701

The mean value of MDA levels in completely edentulous patients was 2.6 ± 1.23 (class I, 2.4 ± 1.07; class II, 2.3 ± 1.83; class III, 2.7 ± 1.27; class IV, 2.3 ± 0.82) (Table 3). The Mann-Whitney test showed no statistically significant difference in MDA levels between class I vs. II, class I vs. III, class II vs. III, class II vs. IV, class III vs. IV and class I vs. IV (p<0.05) (Table 4).

**Table 3:** Mean values of MDA and SOD levels among the four classes MDA, malondialdehyde; SOD, superoxide dismutase

		Class				
		I	II	III	IV	Total
MDA levels (µM/ml)	N	11	10	14	10	45
	Mean	2.4	2.3	2.7	2.3	2.5
	SD	1.07	1.83	1.27	0.82	1.23
SOD levels (units/ml)	Valid N	11	10	14	10	45
	Mean	1.4	1.9	2.0	1.8	1.8
	SD	0.70	0.35	0.00	0.42	0.47

**Table 4:** Mann-Whitney test with Bonferroni correction for pairwise comparison in relation to class MDA, malondialdehyde; SOD, superoxide dismutase

	Class	p-value
MDA levels	I vs. II	0.818
	I vs. III	0.536
	I vs. IV	0.743
	II vs. III	0.550
	II vs. IV	0.963
	III vs. IV	0.305
SOD levels	I vs. II	0.096
	I vs. III	0.004
	I vs. IV	0.148
	II vs. III	0.186
	II vs. IV	0.680
	III vs. IV	0.087

The Mann-Whitney test showed a statistically significant difference in MDA levels between denture wearers and non-denture wearers (p<0.05) whereas no statistically significant difference was found in SOD levels between denture wearers and non-denture wearers (Table 5).

**Table 5:** Mann-Whitney test to compare MDA and SOD levels between denture wearers and non-denture wearers MDA, malondialdehyde; SOD, superoxide dismutase

Variables	Previously denture wearer	N	Mean rank	p-value
MDA levels	No	26	29.29	0.041
	Yes	19	20.90	
SOD levels	No	26	26.32	0.771
	Yes	19	25.50	

The mean values of MDA and SOD levels in completely edentulous patients in males and females are shown in Table 6. The Mann-Whitney test showed no statistically significant difference in MDA and SOD levels in males and females (p<0.05).

**Table 6:** Mann-Whitney test to compare MDA and SOD levels between males and females MDA, malondialdehyde; SOD, superoxide dismutase

	Gender	N	Mean	SD	p-value
MDA levels	Male	26	2.4	1.25	0.175
	Female	19	2.9	1.35	
SOD levels	Male	26	1.8	0.39	0.977
	Female	19	1.8	0.52	

## Discussion

Residual ridge resorption is a sequela of tooth loss and is a constant phenomenon decreasing the size of the ridges. The rehabilitation of edentulous patients with increased RRR is usually a challenging procedure. The characteristic feature of the residual bony architecture is that it undergoes lifelong catabolic remodelling. This phenomenon starts from the point of tooth extraction and continues throughout life. Following extraction, a cascade of inflammatory mediators is initiated and subsequently plays a role in RRR. There will be always a difference in the amount of resorption between the maxilla and the mandible as the surface area of the mandible is less compared to the maxilla. Kovacić et al. showed that RRR is 2.5 times more in the mandible than the maxilla after a one-year period. A higher rate of RRR was recorded at frontal sites compared to lateral sites of both maxillary and mandibular residual ridges [8]. The rate of resorption varies between each individual and within the same individual over time and site [1,9]. This study aimed at finding the correlation between oxidative stress and antioxidants in RRR in completely edentulous patients using the biomarkers MDA and SOD.

The oxidative damage of macromolecules increases the generation of ROS. The release of ROS plays a vital role in inflammation and disease development. One of the methods of studying this mechanism is evaluating the by-products released through this mechanism. MDA is the principal and most studied product in saliva and gingival crevicular fluid (GCF), and is considered a good indicator of oxidative damage of the bone in the form of bone resorption through lipid peroxidation. Neilsen et al. showed MDA as a biomarker of oxidative stress [10]. In the present study, MDA and SOD were evaluated in completely edentulous patients based on the classification of edentulous structures. There was no statistically significant difference in MDA levels whereas SOD levels showed statistical significance among all four classes (p<0.05). It reflects that even though the amount of tissue damage in the form of resorption does not vary among different classes, the antibody defense is more in patients with more resorption. This is in correlation with the results of the study by Panjamurthy et al., Akalin et al. and Zaidan who showed higher levels of MDA and SOD [11-13]. Despite the fact that no previous studies on edentulous patients have been conducted, this study correlates to elevated levels of periodontitis and bone diseases. Canakci et al. showed lower levels of SOD in patients with chronic periodontitis compared to healthy controls, which is not accordance with the present study [14]. Also, studies done by Brock et al., Karatas et al. and Sculley et al. showed lower activities of SOD [3,15,16]. This can be due to differences in local factors associated with dentulous patients with periodontitis.

Statistically significant MDA levels were seen between groups with ≤1 yr and 5-10 yrs of edentulism period. MDA levels showed a greater increase in the early period of edentulism, which could indicate increased ridge resorption. This is in accordance with the results of Carlsson and Persson who stated that resorption was higher at a rate of 0.13 mm/yr in the first year of edentulism and slowed down to 0.5 mm/yr [9]. Kovacić et al. showed a significantly higher alveolar bone height decrease in those patients who had lost the last remaining teeth more recently [8].

Several longitudinal studies indicate that well-fitting dentures preserve the alveolar ridge. Dentures may help to preserve the buccal plate of the bone when inserted immediately after extraction or at least up to 20 wks or up to 3 yrs [9,17]. Wictorin and Johnson in their studies showed that immediate denture wearing helps in reducing RRR [18-20]. There seems to be a correlation between progressive irreversible bone loss and placement of dentures following extraction. Studies done by Tallgren, Nicol et al., Bergman and Carlsson and Atwood and Coy showed that there was no statistically significant relation between denture wearing and the rate of RRR [1,21-23]. Studies done by Campbell and Carlsson and Persson showed that intensive denture wearing leads to increased RRR [9,24]. Nedelman et al. in a study showed that an inflammatory infiltrate was seen in both denture wearers and non-denture wearers with a higher amount of inflammatory cells in non-denture wearers, concluding dentures protect the underlying mucosa from any injury [25]. These results are in correlation with our study results where there was an increase in both MDA and SOD levels in non-denture wearers with a statistically significant difference in MDA values. This could possibly imply that there was less oxidative damage or prevention of resorption by dentures in these patients. As denture prevents resorption and more bone resorption is seen within one year after extraction, denture placement immediately after extraction as early as possible may help to prevent bone resorption.

Canakci et al. showed higher levels of MDA in patients with chronic periodontitis compared to healthy controls [14]. Guentsch et al. showed higher lipid peroxidation with higher MDA levels and lower antioxidant activity with lower GPx levels [26]. Canakci et al. showed higher levels of serum MDA levels and lower levels of serum and GCF SOD levels [27]. All these study results are in accordance with our present study results concluding that MDA and SOD levels may increase in patients under oxidative stress.

There was no statistically significant difference in MDA and SOD levels with respect to age and gender. MDA and SOD values found in our study correlate with the studies of RRR done using radiographs and other clinical parameters implicating that MDA and SOD may be used as biomarkers of RRR.

One of the limitations of this study is the sample size that was small to conclude the results as statistically significant. Another limitation is that biomarkers assessed in this study (salivary MDA and SOD) reflect the local phenomenon that may be appropriate to assess RRR, but total systemic levels of these biomarkers will depend on several other factors and affect the levels. So future research should include larger sample sizes and focus on the correlation of local and systemic biomarkers together that would provide strong evidence to conclude the relationship between oxidative stress and antioxidants in the RRR phenomenon.

## Conclusions

Residual ridge resorption is the most common debilitation problem in completely edentulous patients. Oxidative stress is part of the ageing process and inflammatory process, and although RRR is considered a chronic, inflammatory process, there can be implications of oxidative stress in RRR. These implications could provide an insight into the possible mechanism and role of oxidative stress and antioxidants in RRR. The results of this study show that MDA and SOD may be used as biomarkers of RRR in completely edentulous patients. The bone resorption is higher up to the first year of edentulism and there may be less resorption of bone in denture wearers.

## References

[1] Bergman B, Carlsson GE (1985). Clinical long-term study of complete denture wearers. J Prosthet Dent.

[2] Lammie GA (1956). Aging changes and the complete lower denture. J Pros Den.

[3] Brock GR, Butterworth CJ, Matthews JB, Chapple IL (2004). Local and systemic total antioxidant capacity in periodontitis and health. J Clin Periodontol.

[4] Hsueh YJ, Chen YN, Tsao YT, Cheng CM, Wu WC, Chen HC (2022). The pathomechanism, antioxidant biomarkers, and treatment of oxidative stress-related eye diseases. Int J Mol Sci.

[5] Ighodaro OM, Akinloye OA (2018). First line defence antioxidants-superoxide dismutase (SOD), catalase (CAT) and glutathione peroxidase (GPX): their fundamental role in the entire antioxidant defence grid. Alexandria Journal of Medicine.

[6] Gönenç A, Ozkan Y, Torun M, Simşek B (2001). Plasma malondialdehyde (MDA) levels in breast and lung cancer patients. J Clin Pharm Ther.

[7] McGarry TJ, Nimmo A, Skiba JF, Ahlstrom RH, Smith CR, Koumjian JH (1999). Classification system for complete edentulism. J Prosthodont.

[8] Kovacić I, Celebić A, Zlatarić DK (2010). Decreasing of residual alveolar ridge height in complete denture wearers. A five year follow up study. Coll Antropol.

[9] Carlsson GE, Persson G (1967). Morphologic changes of the mandible after extraction and wearing of dentures. A longitudinal, clinical, and x-ray cephalometric study covering 5 years. Odontol Revy.

[10] Nielsen F, Mikkelsen BB, Nielsen JB, Andersen HR, Grandjean P (1997). Plasma malondialdehyde as biomarker for oxidative stress: reference interval and effects of life-style factors. Clin Chem.

[11] Panjamurthy K, Manoharan S, Ramachandran CR (2005). Lipid peroxidation and antioxidant status in patients with periodontitis. Cell Mol Biol Lett.

[12] Akalin FA, Baltacioğlu E, Alver A, Karabulut E (2007). Lipid peroxidation levels and total oxidant status in serum, saliva and gingival crevicular fluid in patients with chronic periodontitis. J Clin Periodontol.

[13] Zaidan TF (2009). The role of lipid peroxidation in the inducation and progression of chronic periodontitis. J Bagh Coll Dentistry.

[14] Canakci CF, Cicek Y, Yildirim A, Sezer U, Canakci V (2009). Increased levels of 8-hydroxydeoxyguanosine and malondialdehyde and its relationship with antioxidant enzymes in saliva of periodontitis patients. Eur J Dent.

[15] Karatas F, Ozates I, Canatan H, Halifeoglu I, Karatepe M, Colakt R (2003). Antioxidant status & lipid peroxidation in patients with rheumatoid arthritis. Indian J Med Res.

[16] Sculley DV, Langley-Evans SC (2003). Periodontal disease is associated with lower antioxidant capacity in whole saliva and evidence of increased protein oxidation. Clin Sci (Lond).

[17] Johnson K (1977). A study of the dimensional changes occurring in the maxilla following closed face immediate denture treatment. Aust Dent J.

[18] Wictorin L (1964). Bone resorption in cases with complete upper denture. A quantitative roentgenographic-photogrammetric study. Acta Radiol Diagn (Stockh).

[19] Johnson K (1977). A study of the dimensional changes occurring in the maxilla following open face immediate denture treatment. Aust Dent J.

[20] Johnson K (1967). A three-year study of the dimensional changes occurring in the maxilla following immediate denture treatment. Aust Dent J.

[21] Tallgren A (1972). The continuing reduction of the residual alveolar ridges in complete denture wearers: a mixed-longitudinal study covering 25 years. J Prosthet Dent.

[22] Nicol BR, Somes GW, Ellinger CW, Unger JW, Fuhrmann J (1979). Patient response to variations in denture technique. Part II: five-year cephalometric evaluation. J Prosthet Dent.

[23] Atwood DA, Coy WA (1971). Clinical, cephalometric, and densitometric study of reduction of residual ridges. J Prosthet Dent.

[24] Campbell RL (1960). A comparative study of the resorption of the alveolar ridges in denture-wearers and non-denture-wearers. J Am Dent Assoc.

[25] Nedelman C, Gamer S, Bernick S (1970). The alveolar ridge mucosa in denture and non-denture wearers. J Pros Dent.

[26] Guentsch A, Preshaw PM, Bremer-Streck S, Klinger G, Glockmann E, Sigusch BW (2008). Lipid peroxidation and antioxidant activity in saliva of periodontitis patients: effect of smoking and periodontal treatment. Clin Oral Investig.

[27] Canakci V, Yildirim A, Canakci CF, Eltas A, Cicek Y, Canakci H (2007). Total antioxidant capacity and antioxidant enzymes in serum, saliva, and gingival crevicular fluid of preeclamptic women with and without periodontal disease. J Periodontol.

